# A prospective study of physical activity and the risk of pancreatic cancer among women (United States)

**DOI:** 10.1186/1471-2407-8-63

**Published:** 2008-02-28

**Authors:** Brook A Calton, Rachael Z Stolzenberg-Solomon, Steven C Moore, Arthur Schatzkin, Catherine Schairer, Demetrius Albanes, Michael F Leitzmann

**Affiliations:** 1 Division of Cancer Epidemiology and Genetics, NCI/NIH, Rockville, MD, USA; 2 Biostatistics Branch, Division of Cancer Epidemiology and Genetics, NCI, Bethesda, MD, USA

## Abstract

**Background:**

Several epidemiologic studies have examined the association between physical activity and pancreatic cancer risk; however, the results of these studies are not consistent.

**Methods:**

This study examined the associations of total, moderate, and vigorous physical activity to pancreatic cancer in a cohort of 33,530 U.S. women enrolled in the Breast Cancer Detection Demonstration Project (BCDDP). At baseline (1987–1989), information on physical activity over the past year was obtained using a self-administered questionnaire. Cox proportional hazards regression was used to estimate relative risks (RR) and 95% confidence intervals of pancreatic cancer risk.

**Results:**

70 incident cases of pancreatic cancer were ascertained during 284,639 person years of follow-up between 1987–1989 and 1995–1998. After adjustment for age, body mass index, smoking status, history of diabetes, and height, increased physical activity was related to a suggestively decreased risk of pancreatic cancer. The RRs for increasing quartiles of total physical activity were 1.0, 0.80, 0.66, 0.52 (95% CI = 0.26, 1.05; p_trend _= 0.05). This association was consistent across subgroups defined by body mass index and smoking status. We also observed statistically non-significant reductions in pancreatic cancer risk for women in the highest quartile of moderate (RR = 0.57; 95% CI = 0.26, 1.26) and highest quartile of vigorous physical activity (RR = 0.63; 95% CI = 0.31, 1.28) compared to their least active counterparts.

**Conclusion:**

Our study provides evidence for a role of physical activity in protecting against pancreatic cancer.

## Background

Few risk factors have been established for pancreatic cancer, the fourth leading cause of cancer death in the United States [1]. Cigarette smoking, diabetes, and obesity have been consistently associated with an increased risk of pancreatic cancer; however, few other modifiable lifestyle factors have been identified that inarguably protect against this highly fatal cancer [2,3]. High glucose levels and low insulin sensitivity characteristic of both obesity and early-stage diabetes may lead to pancreatic cell damage and subsequently, an increased risk of pancreatic cancer [4,5]. Physical activity improves insulin sensitivity [6], independent of adiposity [7] and could therefore represent an independent protective factor for pancreatic cancer risk.

Three prospective cohort studies [8-10] and two case-control studies [11,12] have observed a significant inverse association between physical activity and pancreatic cancer; however, eleven other observational studies have found no relationship between the two [13-23]. Among studies reporting an inverse association, the intensity of physical activity related to a lower risk of pancreatic cancer has varied widely. Of the five inverse studies, three suggest the association is limited to vigorous activity [8,10], one observed a lower risk of pancreatic cancer only for moderate activity or walking/hiking [9], and one study detected an inverse association with "regular physical exercise" [12]. Given these diverse findings, further research into the potentially complex relationship between physical activity and pancreatic cancer is warranted. In the present investigation, we evaluated the association between moderate, vigorous, and total physical activity and pancreatic cancer risk in a prospective cohort of women enrolled in the Breast Cancer Detection and Demonstration Project (BCDDP) Follow-up Study.

## Methods

### Study Population

**Figure 1:**
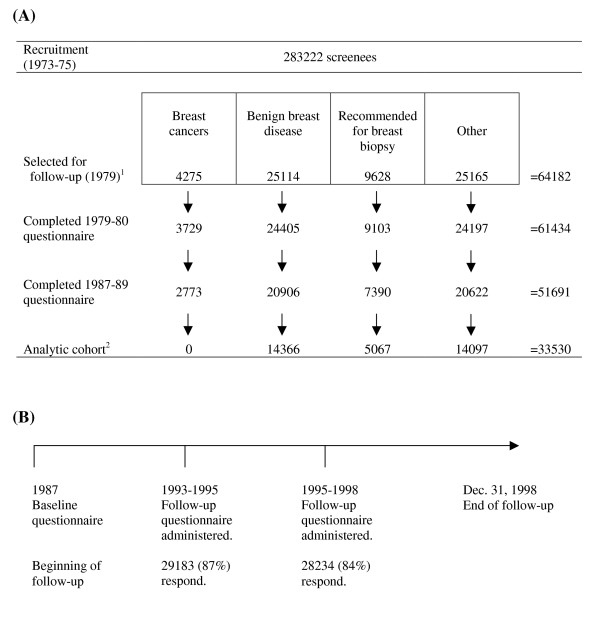
**Participant Flow in the BCDDP Follow-Up Study: **(A) Inclusion criteria for the analytic cohort and (B) Timeline of follow-up for participants in the analytic cohort. ^1^From the original screening population, the BCDDP follow-up cohort included all 4,275 women diagnosed with breast cancer, all 25,114 women with biopsies indicating benign breast disease, all 9,628 women who were recommended for breast biopsy or surgery but did not undergo either procedure, and 25,165 participants (who neither underwent nor were recommended for breast biopsy) matched to women with breast cancer or positive biopsies for breast cancer on age, time of entry into the screening program, length of cohort participation, ethnicity, and location. ^2 ^We excluded 5,691 subjects with a cancer (other than non-melanoma skin cancer) diagnosed prior to the 1987 questionnaire, 10 women who were lost to follow-up, 4,218 women with a missing or extreme body mass index (greater than 3 standard deviations above or below the mean), and 8,242 women with inadequate physical activity information.

Participants were members of the Breast Cancer Detection and Demonstration Project (BCDDP), a mammography screening program sponsored by the National Cancer Institute (NCI) and the American Cancer Society. Between 1973 and 1980, a total of 283,222 cohort participants underwent breast examination at 29 screening centers in 27 U.S. cities. In 1979, a follow-up study consisting of 64,182 of the original cohort members was initiated (Figure 1). The follow-up cohort included all 4,275 women diagnosed with breast cancer, all 25,114 women with biopsies indicating benign breast disease, all 9,628 women who were recommended for breast biopsy or surgery but did not undergo either procedure, and 25,165 participants (who neither underwent nor were recommended for breast biopsy) matched to women with breast cancer or positive biopsies for breast cancer on age, time of entry into the screening program, length of cohort participation, ethnicity, and location. Follow-up questionnaires designed to obtain demographic information, update previously reported exposures to potential risk factors, and identify new cancer diagnoses were mailed to cohort members in 1987, 1993, and 1995. The BCDDP follow-up study was approved by the Institutional Review Board of the National Cancer Institute, and informed consent was obtained from all participants.

For the current analysis, we focused upon the 51,691 participants (84%) who responded to the 1987 questionnaire that first requested information on physical activity, smoking, and diet. Of these 51,691 participants, we excluded 5,691 subjects with a cancer (other than non-melanoma skin cancer) diagnosed prior to the 1987 questionnaire, 10 women lost to follow-up, 4,218 women with a missing or extreme body mass index (greater than 3 standard deviations above or below the mean), and 8,242 women with inadequate physical activity information. A total of 33,530 women were available for analysis in the final analytic cohort.

### Case Ascertainment

A total of 70 cases of incident pancreatic cancer were ascertained during follow-up. Of these 70 cancer cases, 57 (81%) were identified through a search of the National Death Index for death certificates issued during the study follow-up period that indicated pancreatic cancer as the primary or contributory cause of death. An additional 9 cases of pancreatic cancer were identified by self-report on follow-up questionnaires and later confirmed by either pathology reports (n = 4) or data from the state cancer registries (n = 5). Lastly, 4 pancreatic cancer cases not identified by self-report or the National Death Index but indicated on pathology reports or the state cancer registries were included in the analysis. Excluding the cases identified by methods other than death certificate or self-report did not materially alter the results (data not shown).

### Physical Activity Assessment

In the 1987 questionnaire, individuals were asked to estimate the number of hours per typical weekday and weekend day during the past year they spent in each of four activity intensity categories: sleeping, light, moderate, and vigorous activity. Participants were instructed that for each day, the total number of hours across their four activity estimates should equal 24 hours. Examples of recreational, occupational, and/or household activities were given for each activity intensity category. Examples of light activity included office work and watching TV while examples of moderate activity included light housework, hiking, and recreational tennis. Several activities, including heavy housework and running, were given as examples of vigorous activity.

To be considered valid, a participant's activity estimates had to fall within the following plausible range of values that were defined by a set of predefined standards: sleep, 4–14 hours; light activity, 0–20 hours; moderate activity, 0–18 hours; and vigorous activity, 0–18 hours. The acceptable range for the total across all four activity categories was 20–28 hours. For a participant's data to be deemed valid, both the number of hours they reportedly spent sleeping and the total across the four activity categories had to fall within the acceptable range. In addition, a participant's estimates for light, moderate, and vigorous activity had to be within the acceptable range or left blank (in which case a zero value was imputed). As mentioned previously, participants whose activity data did not meet these criteria or who did not provide any information on physical activity were excluded from the analytic cohort.

To generate a physical activity index (PAI), the reported hours in each activity category were first proportioned to total 24 hours per day. This adjustment was performed separately on both the weekday and weekend day activity data. These adjusted values were then used to create two physical activity indices (one for weekday and one for weekend day activity) based on the metabolic equivalent task units (METS) set for each activity intensity category (one MET is defined as the energy expended while sitting quietly, equal to 3.5 ml O_2_/kg/min)[24]. The following formula was used to calculate the PAI for a typical weekday or weekend day.

PAI for a usual day = (hours of sleep * 1 MET) + (hours of light physical activity * 2 METS) + (hours of moderate physical activity * 4 METS) + (hours of vigorous activity * 6 METS)

The two PAI estimates, one for weekday activity and the other for weekend day activity, were used to generate a weighted estimate of average daily total activity expressed in MET units and calculated as follows:

PAI (average MET-hours/day) = [(weekday PAI * 5 days) + (weekend day PAI * 2 days) ]/7 days

In addition to total physical activity as estimated by the PAI, the number of hours spent engaged in vigorous and moderate activity were considered separately as individual measures of physical activity.

Although our physical activity questionnaire has not been compared to activity diaries or another similar validation tool, our physical activity instrument predicts cardiovascular mortality in our cohort (data not shown). In addition, our instrument resembles that of the Framingham Heart Study [25] and a similar instrument that was a key component of the College Alumnus Physical Activity Questionnaire (PAI-CAQ) [26]. In each of these questionnaires, participants are asked to report time spent per day in sleep, light activity, moderate activity, and heavy or vigorous activity and this data is subsequently used to calculate a summary physical activity index. The Framingham and PAI-CAQ questionnaires have demonstrated significant correlations between physical activity and reference measures such as indirect calorimetry in the former [25] and maximum oxygen intake, percentage body fat, high-density lipoprotein levels, and body mass index in the latter [27,28].

### Statistical Analysis

Cox proportional hazards regression was used to estimate the relative risk (RR) and 95% confidence intervals (CI) of pancreatic cancer. The number of person-years each participant contributed to the cohort (defined as the number of years between completion of the 1987 questionnaire and their study exit date) served as the underlying time metric. Participants exited the study at the time of pancreatic cancer diagnosis, death from any cause, or completion of the final follow-up questionnaire (1995–1998), whichever came first. The exit date for a participant who was lost to follow-up was assigned as the date of last contact during 1995 and 1998 or, if the individual could not be contacted, the date of their last completed questionnaire plus the mean time between completion of successfully completed questionnaires derived from the cohort as a whole. The proportional hazards assumption was tested using a cross-product term that included the physical activity variable of interest and follow-up time in person-years. The proportional hazards assumption was satisfied for total, vigorous, and moderate physical activity and for all covariates (all p_interaction _> 0.05).

Total, moderate, and vigorous physical activity was analyzed separately for its association with pancreatic cancer. Total physical activity (measured by the PAI) was categorized into quartiles of 34.0–50.1, 50.11–56.59, 56.6–63.43, and 63.44–100.2 MET-hours/day. For the average daily hours spent in moderate activity, quartiles of 0–3.71, 3.72–6.0, 6.01–8.0, and 8.01–18.0 hours/day were generated. Given the high proportion (38%) of participants who reported engaging in no vigorous activity, individuals reporting zero hours of vigorous activity per day were placed into one category. Tertiles of daily average hours of vigorous activity among those reporting any vigorous activity were then created to form a total of four categories (0, 0.1–1, 1.1–2, 2–12 hours/day).

All covariate information was obtained from the 1987 questionnaire. We evaluated the association between physical activity and pancreatic cancer risk in two models. In the first model, we adjusted for age only (continuous) while in the second model, we additionally adjusted for potential risk factors for pancreatic cancer including height (continuous), body mass index (continuous), race (Caucasian, Hispanic/Hispanic origin, Black, Japanese/Chinese/Other Asian, Other/Unknown), smoking status (never smoker, current smoker (≤ 30 pack-years), current smoker (> 30 pack-years), former smoker (quit smoking ≤ 14 years before baseline), former smoker (quit > 14 years prior)), and personal history of diabetes (yes/no). Moderate and vigorous activities were mutually adjusted for one another in their respective models. To test for a linear trend across increasing quantiles of physical activity, the median of each physical activity quartile or category was entered as a single ordinal variable, the coefficient of which was evaluated by the Wald test. Effect modification was assessed by creating cross-product interaction terms between physical activity and covariates included in the multivariable model. The Wald test was used to evaluate the statistical significance of each cross-product interaction term. All P-values were based on two-sided tests, and all statistical analyses were conducted using SAS release 8.01 (SAS Institute, Cary, NC).

## Results

During 284,639 person years of follow-up between 1987 and 1998, 70 incident cases of pancreatic cancer were identified. The median number of hours reported by cohort participants of light, moderate, and vigorous activity proportioned to 24 hours were 9.0, 6.0 and 0.7 hours, respectively.

Direct age-standardized baseline characteristics of the cohort by quartile of PAI are shown in Table 1. Physically more active women had a lower BMI, tended to smoke less, and were less likely to report education greater than high school or a history of diabetes than women who were less physically active. The mean age at entry into the study among cohort participants was 61.2 (+/- 7.8 SD).

**Table 1:** Baseline characteristics of the BCDDP Follow-up Study participants (n = 33,530) by quartile of total physical activity ^1,2^

**Characteristic**	**PAI Quartile (MET-hours/day)**			
	**Q1 34.0–50.1**	**Q2 50.11–56.59**	**Q3 56.6–63.43**	**Q4 63.44–100.2**
Number of participants	8382	8450	8345	8353
Physical activity (median hours/day)				
Sleep	7.8	7.7	7.5	7.2
Light	13.6	10.2	8.0	5.3
Moderate	2.4	5.5	7.3	8.6
Vigorous	0.2	0.6	1.2	2.9
Age (years)	61.6	61.3	60.9	61.0
Body mass index (kg/m^2^)	25.0	24.7	24.5	24.3
Personal history of diabetes mellitus (%)	0.06	0.04	0.03	0.04
Smoking status (%)				
Never smoker	0.53	0.55	0.57	0.58
Current smoker (< = 30 pack-years)	0.06	0.07	0.06	0.08
Current smoker (> 30 pack-years)	0.07	0.06	0.05	0.05
Former smoker (quit < = 14 years prior)	0.14	0.13	0.13	0.11
Former smoker (quit > 14 years prior)	0.19	0.18	0.17	0.16
Education (> high school; %)	54.8	47.7	46.8	42.8
Race (% white)	89.8	90.1	89.7	87.3

^1 ^Means unless noted otherwise. ^2 ^All values (except age) were directly standardized to the age distribution of the cohort.

In age-adjusted analysis, we observed a significant inverse dose-response relationship between increasing levels of total physical activity, measured by PAI, and pancreatic cancer risk (p_trend _= 0.04) (Table 2). Additional adjustment for height, body mass index, smoking status, and history of diabetes resulted in only slight attenuation of the test for trend (p_trend _= 0.05). Exclusion of the first three years of follow-up had little effect on risk estimates (p_trend _= 0.08). We also examined the physical activity and pancreatic cancer relation among women 65 years of age or younger and women older than 65 years of age. The direction of the physical activity and pancreatic cancer association did not vary by age group nor were there major differences in tests of linear trend (P_trend _= 0.09 and 0.28, for each respective age group; P_interaction _= 0.64).

**Table 2:** Relative risk (RR) of pancreatic cancer in relation to physical activity in the BCDDP Follow-up Study

***Total Physical Activity *^1^**					*p-trend*
MET-hours/day	34.0–50.1	50.11–56.59	56.6–63.43	63.44–100.2	
Cases	24	19	15	12	
Person-years	70137	72150	71650	71701	
Age-adjusted RR (95% CI)	1.00	0.79 (0.43, 1.44)	0.64 (0.34, 1.23)	0.51 (0.25, 1.02)	0.04
Multivariate RR (95% CI) ^2^	1.00	0.80 (0.44, 1.47)	0.66 (0.34, 1.26)	0.52 (0.26, 1.05)	0.05
***Moderate Activity *^1,3^**					
Hours/day	0–3.71	3.72–6.0	6.01–8.0	8.01–18.0	
Cases	17	25	18	10	
Person-years	71921	69238	65778	78703	
Age-adjusted RR (95% CI)	1.00	1.73 (0.92, 3.25)	1.32 (0.67, 2.60)	0.56 (0.26, 1.23)	0.14
Multivariable RR (95% CI)^2,4^	1.00	1.79 (0.95, 3.36)	1.36 (0.69, 2.69)	0.57 (0.26, 1.26)	0.16
***Vigorous Activity *^5,6^**					
Hours/day	0	0.1–1.0	1.1–2.0	2.1–12.0	
Cases	34	15	11	10	
Person-years	108041	66876	52061	57661	
Age-adjusted RR (95% CI)	1.00	0.88 (0.47, 1.63)	0.83 (0.41, 1.66)	0.65 (0.32, 1.33)	0.23
Multivariate RR (95% CI) ^2,7^	1.00	0.90 (0.48, 1.68)	0.80 (0.40, 1.58)	0.63 (0.31, 1.28)	0.18

^1 ^Categorized into quartiles. ^2 ^The multivariable model is adjusted for age, body mass index, race, smoking status, history of diabetes, and height. ^3 ^On the 1987 questionnaire, the following were provided as examples of moderate activity: light housework, vacuuming, washing clothes, painting, home repairs, lawn mowing, general gardening, raking, light sports or exercise, walking, hiking, light jogging, recreational tennis, bowling, golf, and bicycling on level ground. ^4 ^Also adjusted for vigorous activity. ^5 ^Vigorous physical activity: category of zero hours per day, followed by tertiles. ^6 ^The following were listed as examples of vigorous activity on the 1987 questionnaire: heavy housework, scrubbing floors, washing windows, heavy yardwork, digging in garden, chopping wood, strenuous sports/exercise, running, fast jogging, competitive tennis, aerobics, bicycling on hills, and fast dancing. ^7 ^Also adjusted for moderate activity.

Removing body mass index from the multivariable model did not materially alter the results (data not shown). The apparent inverse association between increasing quartiles of PAI and pancreatic cancer risk was consistent across subgroups defined by age, height, body mass index, smoking status, and diabetes status (all p_interaction _> 0.05). Comparing women at the extremes of PAI, the RRs of pancreatic cancer were similar among women with a body mass index greater than or equal to 25 kg/m^2 ^(27 pancreatic cancer cases; RR = 0.33; 95% CI = 0.09, 1.18) and those with a body mass index less than 25 kg/m^2 ^(43 pancreatic cancer cases; RR = 0.67; 95% CI = 0.29, 1.56) (p_interaction _= 0.61).

The results of separate analyses examining the risk of pancreatic cancer according to quartiles of moderate and categories of vigorous activity are also presented in Table 2. Increasing level of vigorous activity and moderate activity was statistically nonsignificantly associated with decreased risk of pancreatic cancer (p_trend _= 0.18 and 0.16, respectively). When women in the highest quartile of moderate activity were compared to a collapsed category containing women in the three lowest quartiles, a significant inverse association for moderate activity was detected (RR = 0.43; 95% CI = 0.22, 0.83).

## Discussion

In this prospective study of U.S. women, high levels of total physical activity appeared to be associated with a lower incidence of pancreatic cancer. Compared to the least physically active women in our cohort, women reporting the highest levels of total physical activity were at a marginally statistically significant, 50 percent lower risk of pancreatic cancer. Our results for the highest quartiles of moderate and vigorous physical activity were also modestly suggestive of an inverse relationship between physical activity and pancreatic cancer risk.

Five previous epidemiologic studies have observed a reduction in pancreatic cancer risk of at least 30 percent with various measures and intensities of recreational [9-12] or occupational [8,10] physical activity. Employing a physical activity measure that did not account for activity intensity, a Japanese nested case-control study reported a relative risk of pancreatic cancer of 0.66 (95% CI = 0.43–1.01) comparing individuals who reported engaging in regular physical activity at least two times per week to those who exercised less than two times weekly [12]. When the analysis was limited to women, the magnitude of risk reduction was greater (RR = 0.55; 95% CI = 0.27–1.10) but did not reach statistical significance, likely due to lower statistical power [12]. In contrast, a Canadian case-control study [11] that included an assessment of activity intensity and used a composite measure of moderate and vigorous recreational physical activity reported a significantly lower risk of pancreatic among active men (RR = 0.53; RR = 0.31–0.90), but not women (RR = 0.80; 95% CI = 0.41–1.54). In the same study [11], neither moderate nor vigorous activity alone was associated with pancreatic cancer for either sex. Differently, in a pooled analysis of data from Harvard's Health Professionals' Follow-up Study and the Nurses' Health Study, high levels of moderate recreational physical activity (RR = 0.45; 95% CI = 0.29, 0.70; p_trend _= < 0.001) but not vigorous activity (RR = 0.91; 95% CI = 0.58–1.42; p_trend _= 0.74) were associated with a reduction in pancreatic cancer risk [9]. In that study, body mass appeared to modify the physical activity-pancreatic cancer relationship such that the greatest degree of protection conferred by physical activity was seen among individuals who were overweight or obese. This observation differs from the present finding of no effect modification by body weight.

The results of a prospective cohort study of male Finnish smokers challenge the findings related to activity intensity from the pooled analysis described immediately above [9], reporting that the protective effect of physical activity on pancreatic cancer risk appeared limited to activity of vigorous, as opposed to moderate, intensity [10]. The same study also suggests that high levels of occupational physical activity (RR = 0.27; 95% CI = 0.15–0.89) confer a greater degree of protection against pancreatic cancer than recreational physical activity (RR = 0.86; 95% CI = 0.43–1.72) [10]. Only one other study to date has observed a significant inverse association between occupational physical activity and pancreatic cancer risk [8].

Nine prospective cohort studies [13,15-21,23] and two case-control studies [14,22] have reported null or positive associations between physical activity and pancreatic cancer risk. Importantly, four of the null studies consisted almost exclusively ^13 ^or entirely [14,20,23] of men and thus, differ from our study of all women, whose physical activity habits and reporting patterns likely differ from those of men. Furthermore, several of the null studies were performed among special populations of longshoremen [20], college alumni [15,20], or professional athletes [23] and consequently, may not be strictly comparable to our study. Additionally, several null studies used relatively crude [8] or surrogate measures of physical activity [14,21] increasing the potential for exposure misclassification. For example, a case-control study of cancer patients in Missouri used job title as a surrogate measure for occupational physical activity level [14] while a Norwegian cohort study used the question "how often do you feel worn out after work?" to estimate physical activity level on the job [18]. Most recently, two Japanese prospective cohort studies used a single question on participation in sports to approximate total recreational physical activity [16,17]. Importantly, neither of these studies were able to examine intensity-specific associations between physical activity and pancreatic cancer risk [16,17].

In the prospective Cancer Prevention Study (CPS)-II Nutrition Cohort, the RR of pancreatic cancer was 1.20 (95% CI = 0.63, 2.27) comparing individuals at the extremes of recreational physical activity [21]. However, when considering physical activity data obtained ten years prior to baseline, the CPS-II study observed a significant 26% reduction in pancreatic cancer risk with moderate physical activity, suggesting that differences in actual versus recalled level of physical activity were partly responsible for the results. In the same study, only 33% of men and 26% of women reported engaging in any moderate to vigorous recreational physical activity. Thus, the intensity of activity among CPS-II cohort members may not have been sufficient to confer protection against pancreatic cancer and could be an additional reason to help explain that study's null findings.

The most recent prospective data on physical activity and pancreatic cancer comes from the Multiethnic Cohort Study [19]. That study reported no relation of physical activity to pancreatic cancer, although the suggestion of an inverse association was noted among women. As compared with the lowest quartile of total activity, women in the highest quartile had a multivariate RR of 0.81 (95% CI = 0.55, 1.20). Much of the apparent inverse association between physical activity and pancreatic cancer was due to the relation with moderate activity. Women engaging in 7 or more hours per week of moderate activity had a RR of 0.68 (95% CI = 0.47, 1.00) as compared to those engaging in less than 1 hour per week of moderate activity. In contrast, the relation with vigorous activity was null [19].

Because physically active individuals may consume a healthier diet and maintain a lower body weight than individuals who are less physically active, the possibility exists that the observed inverse association in our study was confounded by unmeasured or unknown healthy lifestyle habits related to physical activity. However, in contrast to previous studies focusing on recreational activity, in which increasing physical activity tends to track with higher education, physical activity was clearly inversely related to education level in our cohort. This covariate pattern suggests that our physical activity instrument may indeed be capturing not only recreational activity, but also occupational and household activity, reducing the likelihood that our results are confounded by a healthy lifestyle.

Our study is one of the first to report on the association between physical activity and pancreatic cancer risk in women using a composite measure of recreational, occupational, and household activity. Very few studies on this topic [13,19] have included household activity, a type of activity that may be particularly relevant to assess in women. The wide range of activity levels reported by our cohort participants and our evaluation of intensity-specific associations between physical activity and pancreatic cancer further strengthens our prospective analysis.

We were unable to examine individual associations between recreational, occupational, and household activity and pancreatic cancer given that our physical activity assessment represented an overall measure of activity we were unable to disaggregate into its individual components. The modest sample of cases limited our ability to assess with great precision the physical activity and pancreatic cancer association in stratified analyses. Other potential limitations of our study include our reliance on self-reported physical activity estimates assessed at a single time point and limited statistical power to examine possible interactions with smoking, body mass, and diabetes.

## Conclusion

In summary, the results of our prospective study of women are consistent with the hypothesis that greater total physical activity is associated with a reduction in pancreatic cancer risk. This inverse association is biologically plausible in light of the previous literature detailing the insulin-lowering and glucose-sensitizing effects of physical activity as well as the potential relationship between hyperinsulinemia and pancreatic cancer risk [2,4,5].

## Competing interests

The author(s) declare that they have no competing interests.

## Authors' contributions

BAC carried out the primary data analysis and drafted the manuscript. RSS participated in the study concept and interpretation of the data. SCM performed final data analysis and revisions to the manuscript. AS, CS, and DA were responsible for the acquisition of data as well as the study concept and design. AS and CS obtained funding. MLF was the primary consultant to BC for data analysis and manuscript writing and participated in the development as well as revisions of the manuscript. All authors read and approved the final manuscript.

## Pre-publication history

The pre-publication history for this paper can be accessed here:


